# Chemical Profiling and Biological Activities on Nepalese Medicinal Plant Extracts and Isolation of Active Fraction of *Nyctanthes arbor-tristis*

**DOI:** 10.1155/2024/5080176

**Published:** 2024-03-14

**Authors:** Anita Khadka, Akash Budha Magar, Khaga Raj Sharma

**Affiliations:** Central Department of Chemistry, Tribhuvan University, Kirtipur, Kathmandu, Nepal

## Abstract

The importance of medicinal plants for the treatment of different diseases is high from the aspects of the pharmaceutical industry and traditional healers. The present study involves nine different medicinal plants, namely, *Neolamarckia cadamba, Nyctanthes arbor-tristis, Pogostemon benghalensis, Equisetum debile, Litsea monopetala, Spilanthes uliginosa, Desmostachya bipinnata, Mallotus philippensis,* and *Phoenix humilis,* collected from Chitwan district of Nepal for biochemical analysis followed by the isolation of active plant fractions from the bioactive plant extract. The methanolic extracts of roots, barks, seeds, seed cover, and the other aerial parts of plants were used for the phytochemical analysis and biological activities. The DPPH (2,2-diphenyl-1-picrylhydrazyl) free radical scavenging assay was adopted to evaluate the antioxidant activity. Antibacterial activity was evaluated using the agar well diffusion method. The antidiabetic activity was studied by the *α*-amylase enzyme inhibition assay. The highest antioxidant activity was observed in extracts of *Nyctanthes arbor-tristis* followed by *Mallotus philippensis (seed cover)*, *Pogostemon benghalensis, Litsea monopetala*, *Phoenix humilis*, *and Neolamarckia cadamba* with IC_50_ values of 27.38 ± 1.35, 32.08 ± 2.81, 32.75 ± 2.13, 33.82 ± 1.07, 40.14 ± 0.93, and 50.44 ± 3.75 *µ*g/mL, respectively. The highest antidiabetic activity was observed in extracts of *Phoenix humilis* followed by *Desmostachya bipinnata and Pogostemon benghalensis* with IC_50_ values of 95.69 ± 6.97, 99.24 ± 12.6, and 106.3 ± 12.89 *µ*g/mL, respectively. The mild *α*-amylase enzyme inhibition was found in extracts of *Nyctanthes arbor-tristis, Spilanthes uliginosa Swartz, Litsea monopetala, and Equisetum debile* showing IC_50_ values of 110.4 ± 7.78, 115.98 ± 10.24, 149.83 ± 8.3, and 196.45 ± 6.04 *µ*g/mL, whereas *Mallotus Philippensis* (seed cover), *Mallotus philippensis* (seed), and *Desmostachya bipinnata* showed weak *α*-amylase inhibition with IC_50_ values of 208.87 ± 1.76, 215.41 ± 2.09, and 238.89 ± 9.27 *µ*g/mL, respectively. The extract of *Nyctanthes arbor-tristis* showed high zones of inhibition against *S. aureus* (ATCC 25923) and *E. coli* (ATCC 25922) of ZOI 26 and 22 mm, respectively. The chemical constituents isolated from the active plant *Nyctanthes arbor-tristis* were subjected to GCMS analysis where the major chemical compounds were 11,14,17-eicosatrienoic acid and methyl ester. These results support the partial scientific validation for the traditional uses of these medicinal plants in the treatment of diabetes and infectious diseases by the people living in different communities of Chitwan, Nepal.

## 1. Introduction

Ayurveda is considered to be the most ancient medicinal system that had developed as a fully fledged medical system during 1000 BC [1]. Many drugs used in modern medicinal practices are derived from the herbal remedies in the history of traditional therapies including opium, aspirin, digitalis, and quinine. In recent years, the use and search for potent natural compounds as drug candidates and dietary supplements have derived from plants. It has been reported that plant-derived chemical compounds have potential against simple to life-threatening diseases, and at least 7,000 medical compounds in modern pharmacopeia are derived from plants [2]. The World Health Organization estimated that about 80% of the world population relies on plant extracts or their active constituents as folk medicine in traditional therapies. However, due to the inaccessibility of modern medicinal practices, a large number of Nepalese people depend upon a wide range of natural medicines for their primary healthcare based on ethnobotanical knowledge [3]. Nepal is rich in natural biodiversity which reflects the floral and faunal variations. This biodiversity supports the livelihood of people (80–90%) regarding the utilization of plants as medicine. Medicinal plants have assumed greater importance in recent days due to the tremendous potential they offer in developing new drugs against many diseases and illnesses that affect human beings. Herbal plants constitute the major constituents of most indigenous medicines, and a large number of medical preparations contain one or more ingredients of plant origin [4]. Plant-derived pharmaceuticals have high demand in the world market as fragrances, flavors, and color ingredients [5].

People of different communities in the world have been using medicinal plants for many years in the treatment of different diseases like cancer, Parkinson, Alzheimer, diabetes, and several infectious diseases [6, 7]. These plants are used as a source of anticancer, antidiabetic, antioxidant, and antimicrobial agents. Antioxidants are compounds that are present at low concentrations but effectively impede or retard the oxidation of substrates [8]. They protect the cell from oxidative damage caused by the reactive oxygen and nitrogen species. An imbalance between antioxidants and reactive oxygen species is the underlying basis of oxidative stress, leading to cellular damage [2, 9].

Compounds of natural, synthetic, and semisynthetic origin that kill or inhibit the growth of microorganisms without little or no damage to the host cell are antimicrobial agents. Traditionally, people of different communities have been using medicinal plant extracts in the crude form as an herbal medicine against several infectious diseases [10]. The chemical compounds that act as the most important weapons against microbial infections in humans are antibiotics. Many commonly used antibiotics have become less and less effective against certain illnesses due to the emergence of drug-resistant bacteria [11]. The scope of antimicrobials from natural sources is in high demand because of the increasing rate of multidrug-resistant strains as well as newly developed strains with reduced susceptibility to available antimicrobial agents [12]. The systematic screening of antimicrobial plant extracts is a continuous effort to find new drug candidates with high activity against multidrug-resistant microorganisms [13].

Diabetes is a life-threatening disease that arises due to the insufficient production of insulin by the pancreas (type 1 diabetes) or the human body's failure to properly use produced insulin (type 2) [14]. Type 2 diabetes has received considerable attention due to its prevalence among the population. One major therapeutic target for antidiabetic drugs is the *α*-amylase and *α*-glucosidase enzymes. *α*-Amylase is a group of endoglucanases secreted by the salivary glands and pancreas. They initiate carbohydrate digestion by hydrolyzing *α*-1,4-glycosidic bonds in carbohydrates to produce oligosaccharides [15, 16]. Oligosaccharides and disaccharides thus produced are cleaved into glucose by *α*-glucosidase enzymes produced near the brush border enterocyte region of the jejunum [17]. Inhibition of *α*-amylase and *α*-glucosidase significantly delays the digestion of carbohydrates and subsequent absorption of glucose [18]. Thus, both *α*-amylase and *α*-glucosidase inhibitors are effective in controlling post prandial hyperglycemia [19]. Compounds bearing more than one type of therapeutic action are found to be effective in the treatment of diabetes [20].

**Figure 1:**
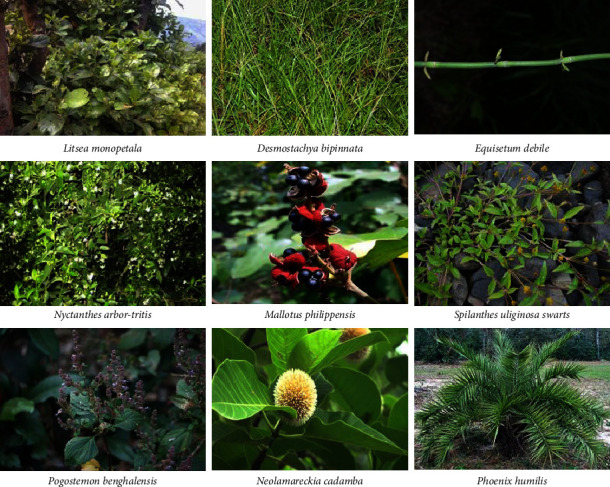
Photographs of the collected plant samples from the study area.

The present study focused on the collection of nine selected medicinal plants from the Chitwan district of Nepal based on their traditional uses (Figure 1). Biochemical profiling of the medicinal plants was established based on phytochemical analysis, antioxidant, antidiabetic, and antibacterial activity giving partial scientific support for using such medicinal plants as antioxidants and antidiabetic and antibacterial agents.

## 2. Materials and Methods

### 2.1. Chemicals and Equipment

The chemicals and reagents used in the experimental section of this research were of analytical grade from Merck, T. Fisher Scientific, and Qualigens chemical companies, India. The major equipment used was Buchi RE111 rotavapor, Thermo Scientific Spectronic 20+ spectrophotometer, grinder, digital weighing machine (GT 210), column (Fortuna W.G.C, *Optifix,* Germany), micropipettes (Erba Biohit), and water bath (Physio Lab Scientific Industries, Ambala Cantt, India).

### 2.2. Collection and Identification of Plants

Plant materials were collected from the Bharatpur and Tandi areas of Chitwan, Nepal. The collected plants were identified at the Central Department of Botany, Tribhuvan University. The plant samples were washed under tap water to remove contaminants such as dust, soil, eggs, and larvae of insects. The clean plant samples were dried in the shade, ground to make powder, and stored in clean and labeled plastic bags. The list of plants collected, parts used, common names, and therapeutic uses are shown in Table 1. The voucher identification number is indicated as AK_1_-AK_10_.

### 2.3. Extraction of Plant Metabolites

The 100 g powder of each plant sample was extracted by cold percolation in methanol (300 mL) at 25°C for 48 hours with frequent agitation. The mixture was filtered through clean cotton. This process was repeated for the complete extraction, and the filtrate was concentrated in a rotary evaporator under reduced pressure at 55°C. The extracts were kept in a beaker, and the percentage yields of the dried plant extracts were calculated. The extracts were stored at 4°C until the biological and chemical analyses were carried out.

### 2.4. Phytochemical Analysis

The preliminary qualitative phytochemical analysis of plant extracts was performed by color differentiation reactions adopting the protocol [30].

### 2.5. DPPH Radical Scavenging Activity

The use of 2,2-diphenyl-1-picrylhydrazyl (DPPH) is a rapid, simple, and inexpensive method to measure the radical scavenging activity by adopting the standard protocol which was previously described by Jamuna et al. [31]. The percentage of DPPH radical scavenging was calculated by using the equation:

(1) $$\begin{array}{c} \text{Radical scavenging}\left(\%\right)=\left[\frac{\left(A_{o}-A_{s}\right)}{A_{o}}\right]*100, \end{array}$$

where *A*_*o*_ is the absorbance of the control DPPH solution and methanol and *A*_*s*_ is the absorbance of the test sample. IC_50_ (50% inhibitory concentration) is the effective concentration of the sample that is required to scavenge 50% of the DPPH free radicals. IC_50_ values were calculated using the linear regression equation graphically with GraphPad Prism 9 software.

### 2.6. In Vitro *α*-Amylase Inhibitory Studies

The *α*-amylase inhibition assay was performed by adopting the standard protocol of Kusano et al. with slight modifications [32]. The excess starch due to *α*-amylase inhibition was measured at 630 nm as a blue, starch-iodine complex. 200 mg starch was dissolved in 25 mL of NaOH (0.4 M) by heating at 100°C for 5 minutes to prepare the substrate. After cooling, the pH was adjusted to 7.0, and the final volume was made to 100 mL by adding distilled water. Acarbose was used as a positive control. 400 *μ*L of test solution was preincubated at 37°C for 5 minutes with 200 *μ*l of acarbose or plant extract with varying concentrations (40, 80, 160, 320, and 640 *μ*g/mL), followed by 200 *μ*L of 50 *μ*g/mL *α*-amylase (20 mM phosphate buffer with 6.7 mM NaCl, pH 6.9), and incubated at 37°C for 15 min. Terminations of the reaction was carried out by adding 800 *μ*L of HCl (0.1 M). After that, 1000 *μ*L of 2.5 mM iodine reagent was added to the mixture, and the absorbance was measured at 630 nm. The triplicate reading was measured by using a spectrophotometer. The *α*-amylase enzyme inhibition was expressed as percent inhibition calculated using the equation given below: The % *α*-amylase inhibition was plotted against the extract concentration, and the IC_50_ values were calculated graphically:

(2) $$\begin{array}{c} \alpha ‐\text{amylase inhibition}=\left(1‐\left[\frac{{\text{Abs}}_{2}‐{\text{Abs}}_{1}}{{\text{Abs}}_{4}‐{\text{Abs}}_{3}}\right]\right)*100. \end{array}$$

Abs_1_ is the absorbance of the incubated mixture containing plant sample, starch, and *α*-amylase, Abs_2_ is the absorbance of the incubated mixture of sample and starch, Abs_3_ is the absorbance of the incubated mixture of starch and *α*-amylase, and Abs_4_ is the absorbance of the incubated solution containing starch.

### 2.7. Antibacterial Activity Agar Well Diffusion Method

Antibacterial activity of the plant extracts was performed using the agar well diffusion method. The antimicrobial activity was evaluated by measuring the zone of inhibition (ZOI) [11]. The bacterial strains were obtained from the Central Department of Microbiology, Tribhuvan University. The strains included a Gram-positive bacterium (*Staphylococcus aureus* ATCC 25923) and a Gram-negative bacterium (*Escherichia coli* ATCC 25922). The organisms to be tested were aseptically touched with the help of an inoculating loop from the primary culture plate. Then, it was transferred into a test tube with 10 mL of sterile liquid media of nutrient broth and incubated overnight at 37°C. The media used in the study were prepared according to the manufacturer's recommendation [33].

The prepared sterile Muller–Hinton Agar (MHA) plates were dried to remove the excess moisture from the surface of the media. The agar plates were prepared by labeling them with the name of the bacteria and the name code of the disc. The inocula of bacteria were transferred into a Petri dish containing solid nutrient media of agar using a sterile swab. The sterile cotton swab was dipped into the prepared inocula and swabbed carefully all over the plates. The plate was rotated through an angle of 60° after each swabbing. Finally, the swab was passed around the edges of the agar surface. The inoculated plates were left to dry for minutes at room temperature with a lid closed. For each incubated media plate, wells were made of 6 mm diameter with the help of sterile cork borer no. 6 and labeled properly. Then, 50 *µ*L of the working solution of the plant extract, DMSO as a negative control, and chloramphenicol as a positive control at the same time in the separate well were loaded. The plates were kept for half an hour with a closed lid so that the extract is completely diffused into the media. The plates were kept in an incubator overnight at 37°C. After 24 hours of incubation, the plates were observed for the presence of inhibition of bacterial growth indicated by a clear zone around the wells. The size of the zone of inhibition was measured, and the antibacterial activity was expressed in terms of the average diameter of the zone of inhibition in millimeters. The absence of the zone of inhibition was interpreted as the absence of bacterial activity. The ZOI was measured with the help of a millimeter ruler, and the mean was calculated [34].

### 2.8. Extraction and Isolation of Compounds

The extract of *Nyctanthes arbor-tristis* bark with promising antioxidant and antimicrobial activity was selected for the isolation of chemical constituents. The shade-dried and powder bark of *N. arbor-tristis* (300 g) was extracted exhaustively with methanol by cold percolation. 30 g of methanol extract was fractionated with hexane, ethyl acetate, and dichloromethane of increasing polarity successively. The ethyl acetate soluble fraction showed good separation with a large number of spots in TLC and was subjected to column chromatography to isolate the pure chemical compounds. A slurry was made by mixing 1.8 g of the ethyl acetate fraction with silica gel and loaded onto a silica gel (200 g, E-Merck, 60–120 mesh) packed column having an internal diameter of 4.5 cm with an adsorbent height of 60 cm. The column was eluted with gradients of ethyl acetate in hexane and MeOH in EtOAc to obtain several fractions. The polarity of the eluent was changed after the TLC report of each fraction. The number of spots observed in TLC was visualized under UV-visible light and an iodine chamber.

### 2.9. Analytic Conditions for FTIR

FTIR is an analytical device used to detect functional groups in the organic compounds providing the covalent bonding information (IRTracer-100 Shimadzu). In FTIR, infrared radiation of about 400 to 4000 cm^−1^ is sent through a sample in which some radiation is absorbed and passed through the sample. The radiation absorbed by the molecule is converted into rotational/vibrational energy that produces a signal at the detector in the form of a spectrum typically from 400 cm^−1^ to 4000 cm^−1^ representing a molecular fingerprint of the sample. The 21 scans were used for the better resolution of the IR spectra. Each chemical compound will produce a unique spectral fingerprint, making FTIR analysis a great tool for chemical identification.

### 2.10. Analytic Conditions for GC

GC analysis was performed on the gas chromatography-mass spectrometer GCMS-QP 2010 under the conditions: injection volume 1 *µ*L with a slit ratio of 1 : 90, helium as a carrier gas with an Rtx-5MS column of dimension 30 m × 0.25 mm × 0.25 *µ*m, and temperature programmed at 50, 150, and 250°C with a hold time of 0.0 and 4.0 min identification was accomplished by comparison of MS with those reported in NIST 08 and FFNSCI.3 libraries [35, 36].

### 2.11. Statistical Analysis

Data were recorded as a mean of three determinations of absorbance for each concentration, from which the linear correlation coefficient (*R*^2^) value was calculated. The data were measured as a mean ± standard deviation (SD). The regression equation is given as *Y* = mx + *c*, where *Y* is the absorbance of extract, *m* is the slope from the calibration curve, *x* is the concentration of extract, and *c* is the intercept. The regression equation was used to calculate the concentration of extracts. All the determinations were conducted at least 3 times (*n* = 3); the statistical mean was calculated with ±SD using Microsoft Excel 2013. Means were compared using one-way ANOVA followed by the Tukey test with SPSS version 29 software. Values with *p* < 0.05 were considered significantly different.

## 3. Results

### 3.1. Phytochemical Analysis

The results of the preliminary qualitative phytochemical analysis are shown in Table 2.

The phytochemical analysis showed positive results for glycosides, alkaloids, and terpenoids. The result shows that terpenoids were present in all the extracts, and reducing sugars and glycosides were present in most of the plant extracts. Flavonoids were present only in *Neolamarckia cadamba*, *Desmostachya bipinnata*, *Nyctanthes arbor-tristis,* and *Phoenix dactylifera.* Coumarin was present only in *Phoenix humilis.*

### 3.2. Antioxidant Property

**Figure 2:**
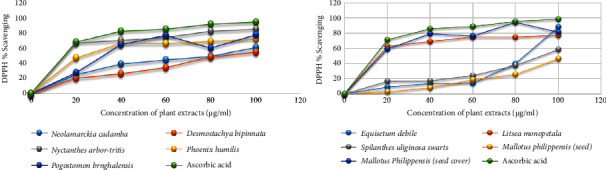
The plot of DPPH radical scavenging against the concentrations of plant extracts.

In this study, the effect on the free radical scavenging ability was determined through the DPPH assay because it is one of the most effective, reactive, reliable, and simple *in vitro* methods. The results of percentage radical scavenging at a different concentration of plant extract with standard ascorbic acid are shown in Figure 2.

The scavenging effects of both plant extracts and the standard on the DPPH radical were expressed as half-maximal inhibitory concentration (IC_50_) values, and the results are reported in Table 3. The linear regression of the percentage of radical scavenging versus concentration was used for the calculation of the concentration of each plant extract required for 50% inhibition of DPPH activity (IC_50_) Table 3. The antioxidant potential has an inverse relation with the IC_50_ value; the lower value of IC_50_ indicates high antioxidant potential. Antioxidants are tremendously important substances that possess the ability to protect the body from damage caused by free radical-induced oxidative stress.

According to the results obtained, *Nyctanthes arbor-tristis, Mallotus philippensis (seed cover), Pogostemon benghalensis,* and *Litsea monopetala* extract showed promising antioxidant activity with the IC_50_ values of 27.38 ± 1.35, 32.08 ± 2.81, 32.75 ± 2.13, and 33.82 ± 1.07 *µ*g/mL. The values were comparable to the IC_50_ values of 24.86 ± 4.6 *µ*g/mL for ascorbic acid used as a positive standard. The plant extract of *Phoenix humilis, Neolamarckia cadamba, Equisetum debile,* and *Desmostachya bipinnata* showed moderate antioxidant activity with IC_50_ values of 40.14 ± 0.93, 78.15, 50.44 ± 3.75, 80.14 ± 4.49, and 81.49 ± 1.59 *µ*g/ml, whereas the plant extracts of *Spilanthes uliginosa Swartz* and *Mallotus philippensis* (seed) impart the poor antioxidant activity with IC_50_ values of 93.41 ± 10.04 and 122.98 ± 6.29 *µ*g/mL, respectively.

### 3.3. Antidiabetic Activity

**Figure 3:**
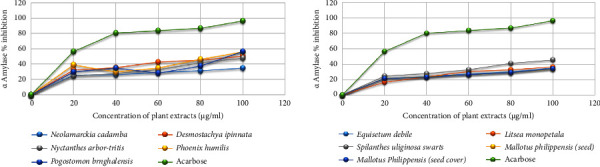
*α*-Amylase inhibition (%) activity of different plant extracts and acarbose at varying concentrations.

The graphical relationship between the *α*-amylase enzyme inhibition and the concentration of plant extracts is shown in Figure 3.

The *α*-amylase enzyme inhibition assay showed that some of the plant extracts had significant inhibitory concentration and found promising *α*-amylase inhibition activity, while the remaining extracts showed mild to poor. The inhibitory concentration (IC_50_) is shown in Table 4.

Many plant extracts have been reported for their antidiabetic activities and are currently being used in ayurveda for the treatment of diabetes. However, there is a lack of scientific validation for using these medicinal plants against diabetes and infectious diseases. In the present study, ten indigenous medicinal plants used against diabetes were screened for their *α*-amylase enzyme inhibition activity. The plant extracts of *Phoenix humilis* and *Desmostachya bipinnata* showed promising *α*-amylase enzyme inhibition activity with IC_50_ values of 95.69 ± 6.97 and 99.24 ± 12.698.54 *µ*g/mL, respectively. The plant extracts of *Pogostemon benghalensis*, *Nyctanthes arbor-tristis*, *Spilanthes uliginosa Swartz*, *Litsea monopetala*, and *Equisetum debile* exhibited moderate *α*-amylase inhibition, whereas *Mallotus philippensis* (seed cover), *Mallotus philippensis* (seed), and *Neolamarckia cadamba* exhibited poor *α*-amylase enzyme inhibition. Acarbose used as a positive standard displayed an IC_50_ value of 28.88 ± 1.4 *µ*g/mL.

### 3.4. Antibacterial Activity

In the present study, the antibacterial activity on ten medicinal plant extracts was recorded against *E. coli* (ATCC 25922) and *S. aureus* (ATCC 25923). The effects shown by the plant extracts against the bacterial strain as zones of inhibition (mm) are listed in Table 5.

The extracts of *Nyctanthes arbor-tristis* and *Mallotus philippensis* (seed cover) showed promising antibacterial activity with the highest ZOI of 26 and 24 mm, respectively, against *S. aureus*. The plant extracts of *Litsea monopetala, Equisetum debile,* and *Phoenix humilis* showed weak antibacterial activity with ZOIs of 14, 12, and 12 mm against *S. aureus*, respectively. The extract of *Nyctanthes arbor-tristis* and *Mallotus philippensis* (seed cover) showed high antibacterial activity against *E. coli* with ZOIs of 22 mm and 14 mm, respectively. The rest of the plant extracts were found to be poor or not active against these organisms.

### 3.5. Isolation of Active Fractions

Based on antioxidant and antimicrobial activities, the bark extract of *Nyctanthes arbor-tristis* was selected as the potent extract for the isolation of active constituents. Different fractions were collected, and purity was checked for each fraction by TLC. Fractions having a similar TLC report were mixed. The list of fractions collected is shown in Table 6.

**Figure 4:**
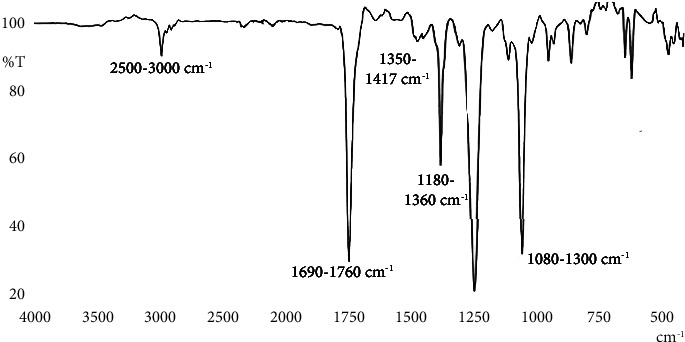
FTIR spectrum of fraction collected in 1% MeOH in EtOAc of *Nyctanthes arbor-tristis*.

Fractions 82–86 were seen as single spots in TLC, but the yield was very low (no significant yield), so the isolation of the compound was not carried out from these fractions. The isolation, characterization, and identification of the compounds are under process. The fractions that show the single spot seen in TLC were subjected to FTIR analysis. FTIR is a useful analytical tool to identify the functional groups in a molecule as each specific chemical band often has a unique energy absorption band. The FTIR spectrum of the fraction collected in 1% MeOH in EtOAc is shown in Figure 4.

The -COOH functional group of carboxylic acid showed IR spectra at 2500–3000 cm^−1^, and the C=O group of ketones, the –CHO group of aldehydes, and the -COOR group of esters showed IR spectra at 1690–1760 cm^−1^. Similarly, the functional group of amines (-NH_2_) showed IR spectra at 1180–1360 cm^−1^, and alcohols (-OH) and ethers (R-O-R′) showed IR spectra at 1080–1300 cm^−1^. It shows the possibility of the presence of compounds having a functional group -COOH, -C=O, -CHO, -COOR, -NH_2,_ -OH, and R-O-R′ in the ethyl acetate fraction collected in 1% MeOH in EtOAc of *N. arbor-tristis*.

### 3.6. GC Analysis

**Figure 5:**
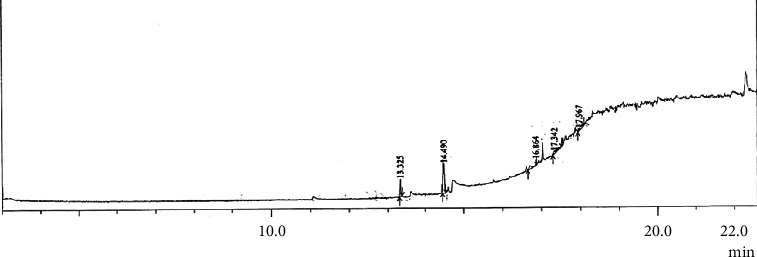
GC chromatogram of fraction collected at 1% MeOH in EtOAc of *Nyctanthes arbor-tristis*.

The fraction collected in 1% MeOH in EtOAc was further subjected to GCMS analysis using a single spot-on TLC report. The gas chromatogram is shown in Figure 5.

The GC/MS analysis of the partially purified fraction of *Nyctanthes arbor-tristis* bark extract search (NIST 08 and FFNSCI.3) revealed the presence of several compounds. The number of peaks was seen in the gas chromatogram out of which 4 compounds were visualized clearly. The major constituents present in the partially purified fraction (1% methanol in ethyl acetate) of *Nyctanthes arbor-tristis* bark were hexadecanoic acid, methyl ester, 11,14,17-eicosatrienoic acid, methyl ester, docosanoic acid, methyl ester, and 2-chloroaniline-5-sulfonic acid. The chemical constituents are shown in Table 7.

## 4. Discussion

The results of the phytochemical analysis showed that plants are rich sources of secondary metabolites. The results of the present study were found to be different from the previously reported results on the phytochemical analysis of some medicinal plants (Ganjewala et al. 2013) [21]. The difference in the phytochemicals as secondary metabolites is due to the variation in altitude of plants, different environmental conditions, method and time of sample collection, extraction procedure, and also due to lab setup and chemical grades [37]. Ascorbic acid can be used as a standard antioxidant showing strong DPPH scavenging activity. Several studies have demonstrated a linear correlation between antioxidant activity and the phenolic content of plant extracts [38, 39]. The results of the present study were compared to the previously reported results of some medicinal plants and found to be almost similar in most of the cases. The antioxidant activity of *N. arbor-tristis* extract was found having an IC_50_ value of 57.93 *µ*g/ml [40]. The same trend for DPPH scavenging activity has been shown in previously reported results of the antioxidant activity (IC_50_) for leaf, flower, root, stem, and fruits of *L. camara* extracts as 16.02 ± 0.94, 28.92 ± 0.19, 31.52 ± 0.74, 46.96 ± 2.51, and 90.11 ± 0.57 *µ*g/ml, respectively. The results are comparable to the results of the present study [41]. Saeidnia et al. reported the *α*-amylase enzyme inhibition activity of butanolic and methanolic extract of *Z. majdae* with IC_50_ values of 24.5 ± 2.1 and 22.0 ± 2.7 mg/mL as compared to acarbose 6.6 ± 3.1 mg/mL. This result was compared to the results of the present study and found that the plant extracts exhibited moderate *α*-amylase enzyme inhibition activity [42]. Khan et al. 2013 reported the antibacterial activity of the cold-water extract of *J. officinale* (leaves) with a ZOI of 16 mm against *P. aeruginosa* followed by *B. subtilis* (13 mm), *E. coli* (7 mm), and *P. vulgaris* (7 mm). Similarly, cold-water extract of *Santalum album* (wood) exhibited a zone of inhibition (15 mm) against *P. vulgaris* followed by *B. subtilis* (14 mm), *E. coli* (10 mm), and *P. aeruginosa* (9 mm). The results of the present study showed that the plant extracts exhibited potent antibacterial activity as compared to the previously reported results [43]. The results of quantitative phytochemical analysis and antioxidant activity in this study were found comparable to the results reported by Bhatt and Dahal in the plant *Elaeocarpus ganitrus* in various solvent extracts of seed from the Ilam District of Nepal [44].

## 5. Conclusion

The present study provides the first pharmacological insight into the antioxidant, antidiabetic, and antibacterial potential of less explored selected medicinal plants from the Chitwan district of Nepal. Phytochemical analysis showed that plant extracts are found to be rich in phenolic and flavonoid content as secondary metabolites. The extracts of *Nyctanthes arbor-tristis, Mallotus philippensis (seed cover), Pogostemon benghalensis,* and *Litsea monopetala* showed high antioxidant capacity, whereas *Phoenix humilis, Neolamarckia cadamba, Equisetum debile,* and *Desmostachya bipinnata* showed moderate antioxidant capacity. These plant extracts showed significant *α*-amylase enzyme inhibition as compared to the most common drug acarbose indicating that the polyphenols and flavonoids present in the extracts reduce postprandial hyperglycemia by delaying carbohydrate digestion. An IC_50_ value of 95.69 ± 6.97 *µ*g/mL observed for *Phoenix humilis* was slightly higher than 28.88 ± 1.4 *µ*g/mL observed for acarbose. The plant extracts of *Nyctanthes arbor-tristis* and *Mallotus philippensis* (seed cover) showed promising antibacterial activity against *E. coli* and *S. aureus*. The results of the present study could be made scientifically more valid by performing the *in vivo* assay in the isolated pure compounds from the active plant extracts selected in this study. The findings of the recent study would be useful for future research directions on the traditionally used medicinal plants in the development of nutraceuticals and pharmaceuticals as drug candidates in the future drug discovery process.

## Figures and Tables

**Table 1:** List of collected plant samples, plant parts used, common names, and therapeutic uses.

Code	Scientific name	Local name	Used parts	Therapeutic uses
AK_1_	*Neolamarckia cadamba*	Kadam	Bark	Antifungal, diuretic, wound healing [21]
AK_2_	*Desmostachya bipinnata*	Kush	Roots	Antibacterial, anticancer [22]
AK_3_	*Nyctanthes arbor tristis*	Parijat	Bark	Rheumatic, ulcer, analgesic, joint pain [23]
AK_4_	*Phoenix humilis*	Thakal	Roots	Sprain, cuts, ulcer [24]
AK_5_	*Pogostemon benghalensis*	Rudilo	Aerial parts	Antifungal, repellent, wound healing [25]
AK_6_	*Equisetum debile*	Hadjorni	Whole plant	Antiaging, hyperpigmentation [26]
AK_7_	*Litsea monopetala*	Kutmiro	Bark	Antioxidant, diarrhea [27]
AK_8_	*Spilanthes uliginosa Swartz*	Marethi	Whole plant	Snakebite, diabetes, weight loss [28]
AK_9_	*Mallotus philippinensis*	Sindoore	Seeds	Coloring agents, skin diseases [29]
AK_10_	*Mallotus philippinensis*	Sindoore	Seed cover	Coloring agent, antioxidant [29]

**Table 2:** Qualitative phytochemical analysis of methanol extract of plant samples.

Groups of compounds	AK_1_	AK_2_	AK_3_	AK_4_	AK_5_	AK_6_	AK_7_	AK_8_	AK_9_	AK_10_
Alkaloids	+	+	+	−	−	−	+	−	−	−
Coumarins	−	−	−	+	−	−	−	−	−	−
Flavonoids	+	+	+	+	−	−	−	−	−	−
Glycosides	−	+	+	+	−	+	+	+	+	+
Polyphenols	−	−	+	−	+	−	−	+	−	−
Quinones	−	−	−	+	+	+	−	+	−	+
Reducing sugars	+	+	+	−	−	+	−	+	−	+
Saponins	+	+	−	+	+	−	−	−	−	−
Terpenoids	+	+	+	+	+	+	+	+	+	+

+The presence and −the absence. AK_1_ = *Neolamarckia cadamba*, AK_2_ = *Desmostachya bipinnata*, K_3_ = *Nyctanthes arbor-tristis*, AK_4_ = *Phoenix humilis*, AK_5_ = *Pogostemon benghalensis*, AK_6_ = *Equisetum debile*, AK_7_ = *Litsea monopetala*, AK_8_ = *Spilanthes uliginosa Swartz*, AK_9_ = *Mallotus philippensis* (seed), and AK_10_ = *Mallotus philippensis* (seed cover).

**Table 3:** DPPH radical scavenging activity as IC_50_.

Plant extracts	Radical scavenging (IC_50_) *µ*g/mL
*Nyctanthes arbor-tristis*	27.38 ± 1.35
*Mallotus philippensis (seed cover)*	32.08 ± 2.81
*Pogostemon benghalensis*	32.75 ± 2.13
*Litsea monopetala*	33.82 ± 1.07
*Phoenix humilis*	40.14 ± 0.93^*∗*^
*Neolamarckia cadamba*	50.44 ± 3.75^*∗∗∗*^
*Equisetum debile*	80.14 ± 4.49^*∗∗∗*^
*Desmostachya bipinnata*	81.49 ± 1.59^*∗∗∗*^
*Spilanthes uliginosa Swartz*	93.41 ± 10.04^*∗∗∗*^
*Mallotus philippensis (seed)*	122.98 ± 6.29^*∗∗∗*^
Standard ascorbic acid	24.86 ± 4.6

Values are mean ± SD; ^*∗*^*p* < 0.5 and ^*∗∗∗*^*p* < 0.01 vs. the positive control (ascorbic acid).

**Table 4:** Inhibitory concentration of plant extracts against the enzyme *α*-amylase.

Plant extracts	*α*-Amylase inhibition activity (IC_50_) *µ*g/mL
*Phoenix humilis*	95.69 ± 6.97^*∗∗∗*^
*Desmostachya bipinnata*	99.24 ± 12.6^*∗∗∗*^
*Pogostemon benghalensis*	106.3 ± 12.89^*∗∗∗*^
*Nyctanthes arbor-tristis*	110.4 ± 7.78^*∗∗∗*^
*Spilanthes uliginosa Swartz*	115.98 ± 10.24^*∗∗∗*^
*Litsea monopetala*	149.83 ± 8.3^*∗∗∗*^
*Equisetum debile*	196.45 ± 6.04^*∗∗∗*^
*Mallotus philippensis* (seed cover)	208.87 ± 1.76^*∗∗∗*^
*Mallotus philippensis* (seed)	215.41 ± 2.09^*∗∗∗*^
*Neolamarckia cadamba*	238.89 ± 9.27^*∗∗∗*^
Acarbose	28.88 ± 1.4

Values are mean ± SD; ^*∗∗∗*^*p* < 0.001 vs. the positive control (acarbose).

**Table 5:** Antimicrobial activity of different plant extracts against pathogenic bacteria.

Plant extracts	Bacteria	ZOI (mm) shown by the extracts at a concentration of 100 mg/mL	ZOI (mm) shown by chloramphenicol as control at 100 mg/mL
*Neolamarckia cadamba*	*E. coli*	—	26
	*S. aureus*	—	35

*Desmostachya bipinnata*	*E. coli*	—	26
	*S. aureus*	—	35

*Nyctanthes arbor-tristis*	*E. coli*	22	26
	*S. aureus*	26	35

*Phoenix humilis*	*E. coli*	11	26
	*S. aureus*	12	35

*Pogostemon benghalensis*	*E. coli*	—	26
	*S. aureus*	—	35

*Equisetum debile*	*E. coli*	9	26
	*S. aureus*	12	35

*Litsea monopetala*	*E. coli*	11	26
	*S. aureus*	14	35

*Spilanthes uliginosa Swartz*	*E. coli*	—	26
	*S. aureus*	—	35

*Mallotus philippensis* (seed)	*E. coli*	—	26
	*S. aureus*	—	35

*Mallotus philippensis* (seed cover)	*E. coli*	14	26
	*S. aureus*	24	35

Negative control = DMSO (no effective antibacterial activity); ZOI = zone of inhibition; *E. coli*: Gram-negative organism; *S. aureus*: Gram-positive organism.

**Table 6:** Isolation of active fractions from the extract of *Nyctanthes arbor-tristis*.

Eluting solvent system	Fraction no	Volume of eluent (mL)	TLC solvent system	TLC report
100% hexane	1–3	300	1% EtOAc in hexane	No spot
100% hexane	4–6	300	1% EtOAc in hexane	No spot
100% hexane	7–10	400	1% EtOAc in hexane	No spot
1% EtOAc in hexane	11–13	300	2% EtOAc in hexane	No spot
1% EtOAc in hexane	14–16	300	2% EtOAc in hexane	No spot
3% EtOAc in hexane	17–20	400	5% EtOAc in hexane	No spot
3% EtOAc in hexane	21–24	400	5% EtOAc in hexane	Tailing
5% EtOAc in hexane	24–28	500	10% EtOAc in hexane	One spot (tailing)
5% EtOAc in hexane	29–33	500	10% EtOAc in hexane	Tailing
5% EtOAc in hexane	34–38	500	10% EtOAc in hexane	Tailing
10% EtOAc in hexane	39–42	400	10% EtOAc in hexane	Tailing
10% EtOAc in hexane	43–46	400	10% EtOAc in hexane	Tailing
15% EtOAc in hexane	47–51	500	20% EtOAc in hexane	No clear spot
25% EtOAc in hexane	52–54	300	30% EtOAc in hexane	Two clear spots
25% EtOAc in hexane	55–58	400	30% EtOAc in hexane	No distinct spot
40% EtOAc in hexane	59–61	300	40% EtOAc in hexane	Tailing
40% EtOAc in hexane	62–64	300	40% EtOAc in hexane	Tailing
60% EtOAc in hexane	65–67	300	70% EtOAc in hexane	Tailing
60% EtOAc in Hexane	68–70	300	70% EtOAc in Hexane	Multi-spots
80% EtOAc in hexane	71–74	400	40% EtOAc in hexane	Tailing
100% EtOAc	75–81	600	1% MeOH in EtOAc	Tailing
1% MeOH in EtOAc	82–86	500	2% MeOH in EtOAc	Single spot
3% MeOH in EtOAc	87–91	500	5% MeOH in EtOAc	One spot (tailing)
5% MeOH in EtOAc	92–96	500	10% MeOH in EtOAc	Single spot (tailing)
10% MeOH in EtOAc	97–101	500	15% MeOH in EtOAc	One spot (tailing)
15% MeOH in EtOAc	102–105	400	25% MeOH in EtOAc	No clear spot
25% MeOH in EtOAc	106–110	500	30% MeOH in EtOAc	Tailing
50% MeOH in EtOAc	111–115	500	60% MeOH in EtOAc	Tailing
75% MeOH in EtOAc	116–120	500	80% MeOH in EtOAc	Tailing
100% MeOH	121–128	700	60% MeOH in EtOAc	Tailing

**Table 7:** Chemical constituents found in the fraction collected in 1% MeOH in EtOAc of *Nyctanthes arbor-tristis*.

Compounds	Retention time (*R*_*t*_)	Area %	Mol. wt	Molecular formula
Hexadecanoic acid, methyl ester	13.325	13.91	270.45	C_17_H_34_O_2_
11,14,17-Eicosatrienoic acid, methyl ester	14.490	47.20	320.51	C_21_H_36_O_2_
Docosanoic acid, methyl ester	16.864	15.34	354.61	C_23_H_46_O_2_
2-Chloroaniline-5-sulfonic acid	17.96	10.76	255.06	C_6_H_4_Cl_2_N_2_O_3_S

## Data Availability

The data that support the findings of this study can be obtained from the corresponding authors on request.

## Abbreviations

DPPH: 2,2′-Diphenyl-1-picrylhydrazyl
IC_50_: The half-maximal inhibitory concentration
ROS: Reactive oxygen species
*µ*g/mL: Microgram per milliliter
mg: Milligram
ZOI: Zone of inhibition
ATCC: American-type culture collection
PPA: Porcine pancreatic amylase
SD: Standard deviation
GCMS: Gas chromatography and mass spectrometry
FTIR: Fourier-transform infrared spectroscopy.

## References

[1] Mukherjee P. K., Harwansh R. K., Bahadur S. (2017). Development of ayurveda– tradition to trend. *Journal of Ethnopharmacology*.

[2] Sharma K. R., Kalauni S. K., Awale S., Pokharel Y. R. (2015). Vitro free radical scavenging activity of methanol extracts of some selected medicinal plants of Nepal. *Journal of Biotechnology and Bioengineering*.

[3] Adhikary P., Roshan K. C., Kayastha D. (2011). Screening and antimicrobial properties of medicinal plants of Dhunkharka community, Kavrepalanchowk, Nepal. *International Journal of Pharmaceutical and Biological Archives*.

[4] Sharma K. R., Rana K. (2020). Biological activities of some selected Nepalese medicinal plants and isolation of chemical constituents from Callicarpa macrophylla. *International Journal of Current Pharmaceutical Research*.

[5] Kunwar R. M., Mahat L., Acharya R. P., Bussmann R. (2013). Medicinal plants, traditional medicine, markets, and management in far-west Nepal. *Journal of Ethnobiology and Ethnomedicine*.

[6] Akbar S. (2020). *Handbook of 200 Medicinal Plants: A Comprehensive Review of Their Traditional Medicinal Uses and Scientific Justifications*.

[7] Ullah R., Alqahtani A. S., Noman O. M. A., Alqahtani A. M., Ibenmoussa S., Bourhia M. (2020). A review on ethno-medicinal plants used in traditional medicine in the Kingdom of Saudi Arabia. *Saudi Journal of Biological Sciences*.

[8] Pisoschi A. M., Pop A., Iordache F., Stanca L., Predoi G., Serban A. I. (2021). Oxidative stress mitigation by antioxidants- an overview on their chemistry and influences on health status. *European Journal of Medicinal Chemistry*.

[9] Aranda-Rivera A. K., Cruz-Gregorio A., Arancibia-Hernández Y. L., Hernández-Cruz E. Y., Pedraza-Chaverri J. (2022). RONS and oxidative stress: an overview of basic concepts. *Oxygen*.

[10] Kose L. S., Moteetee A., Van Vuuren S. (2021). Ethnobotany, toxicity and antibacterial activity of medicinal plants used in the Maseru District of Lesotho for the treatment of selected infectious diseases. *South African Journal of Botany*.

[11] Cavaliers S. J., Rankin I. D., Harbeck R. J., Sautter R. L. (2005). *Manual of Antimicrobial Susceptibility Testing*.

[12] Subba B., Basnet P. (2014). Antimicrobial activity of some medicinal plants from east and central part of Nepal. *International Journal of Applied Sciences and Biotechnology*.

[13] Chan B. C.-L., Lau C. B.-S., Jolivalt C., Chan L., Lau S. (2011). Chinese medicinal herbs against antibiotic-resistant bacterial pathogens. *Science Against Microbial Pathogens: Communicating Current Research and Technological Advances*.

[14] Thant T. M., Aminah N. S., Kristanti A. N., Ramadhan R., Aung H. T., Takaya Y. (2019). Antidiabetes and Antioxidant agents from Clausena excavata root as medicinal plant of Myanmar. *Open Chemistry*.

[15] Narita Y., Inouye K. (2015). Inhibition of porcine pancreas *α*-amylase by chlorogenic acids from green coffee beans and cinnamic acid derivatives. *Coffee in Health and Disease Prevention*.

[16] Najafian M. (2015). The effects of curcumin on alpha amylase in diabetics rats. *Zahedan Journal of Research in Medical Sciences*.

[17] Lebovitz H. E. (1997). Alpha-glucosidase inhibitors. *Endocrinology and Metabolism Clinics of North America*.

[18] Hullatti K., Telagari M. (2015). In-vitro *α*-amylase and *α*-glucosidase inhibitory activity of Adiantum caudatum Linn. and Celosia argentea Linn. extracts and fractions. *Indian Journal of Pharmacology*.

[19] Poovitha S., Parani M. (2016). In vitro and in vivo *α*-amylase and *α*-glucosidase inhibiting activities of the protein extracts from two varieties of bitter gourd (Momordica charantia L.). *BMC Complementary and Alternative Medicine*.

[20] Konya H., Miuchi M., Konishi K. (2009). Pleiotropic effects of mitiglinide in type 2 diabetes mellitus. *Journal of International Medical Research*.

[21] Ganjewala D., Tomar N., Gupta A. K. (2013). Phytochemical composition and antioxidant properties of methanol extracts of leaves and fruits of*Neolamarckia cadamba*(roxb.). *Journal of Biologically Active Products from Nature*.

[22] Sathasivampillai S. V., Sebastian Rajamanoharan P. H. R. (2021). Bioactivities of Desmostachya bipinnata (L.) stapf. *Plant Biotechnology Persa*.

[23] Hiremath V., Hiremath B. S., Mohapatra S., Kumar Das A. (2016). Literary review of Parijata (Nyctanthus Arbor-Tristis Linn.) an herbal medicament with special reference to ayurveda and botanical literature. *Biomedical and Pharmacology Journal*.

[24] Jain P., Jain S., Sharma S., Paliwal S. (2018). Diverse application of phoenix sylvestris: a potential herb. *Agriculture and Natural Resources*.

[25] Shigwan A. V., Khade A. B., Hatpakki B. C., Ghurghure S. M. (2013). A comprehensive review on Pogostemon benghalensis (burm. F.) O. Kuntze research and reviews. *Journal of Pharmacognosy and Phytochemistry*.

[26] Phanit T., Jiaranaikulwanitch J., Phongpradist R. (2023). Cosmeceutical potentials of Equisetum debile Roxb. Ex vaucher extracts. *Applied Sciences*.

[27] Kamle M., Mahato D. K., Lee K. E. (2019). Ethnopharmacological properties and medicinal uses of Litsea cubeba. *Plants*.

[28] Uraku A. J., Ogbanhi M. E. (2015). Nutritional and phytochemical potential of Spilanthes uliginosa (Sw) leaves. *American Journal of Phytomedicine and Clinical Therapeutics*.

[29] Gangwar M., Goel R. K., Nath G. (2014). Mallotus philippinensis Muell. Arg (Euphorbiaceae): ethnopharmacology and phytochemistry review. *BioMed Research International*.

[30] Panchal P., Parvez N. (2019). Phytochemical analysis of medicinal herb (Ocimum sanctum). *International Journal of nanomaterials, Nanotechnology and Nanomedicine*.

[31] Jamuna S., Paulsamy S., Karthika K. (2012). Screening of in vitro antioxidant activity of methanolic leaf and root extracts of Hypochaeris radicata L. (Asteraceae). *Journal of Applied Pharmaceutical Science*.

[32] Kusano R., Ogawa S., Matsuo Y., Tanaka T., Yazaki Y., Kouno I. (2011). *α*-Amylase and lipase inhibitory activity and structural characterization of acacia bark proanthocyanidins. *Journal of Natural Products*.

[33] Bhavyasree P. G., Xavier T. S. (2020). Green synthesis of Copper Oxide/Carbon nanocomposites using the leaf extract of Adhatoda vasica Nees, their characterization and antimicrobial activity. *Heliyon*.

[34] Moond M., Singh S., Sangwan S. (2023). Phytofabrication of silver nanoparticles using Trigonella foenum-graceum L. leaf and evaluation of its antimicrobial and antioxidant activities. *International Journal of Molecular Sciences*.

[35] Nist (2023). Mass spectrometry data center. https://chemdata.nist.gov/.

[36] Mondello L. (2016). *Flavour and Fragrance Natural and Synthetic Compounds GC/MS Library*.

[37] Li Y., Kong D., Fu Y., Sussman M. R., Wu H. (2020). The effect of developmental and environmental factors on secondary metabolites in medicinal plants. *Plant Physiology and Biochemistry*.

[38] Pensec F., Szakiel A., Pączkowski C. (2016). Characterization of triterpenoid profiles and triterpene synthase expression in the leaves of eight Vitis vinifera cultivars grown in the Upper Rhine Valley. *Journal of Plant Research*.

[39] Budha Magar A., Shrestha D., Pakka S., Sharma K. R. (2023). Phytochemistry, biological, and toxicity study on aqueous and methanol extracts of Chromolaena odorata. *The Scientific World Journal*.

[40] Riya (2007). Antibacterial activities and phytochemical analysis of different plant parts of Nyctanthes arbor-tristis (linn.). *Research Journal of Phytochemistry*.

[41] Mahdi-Pour B., Jothy S. L., Latha L. Y., Chen Y., Sasidharan S. (2012). Antioxidant activity of methanol extracts of different parts of Lantana camara. *Asian Pacific Journal of Tropical Biomedicine*.

[42] Saeidnia S., Mirshafie B., Mokhber-Dezfouli N., Manayi A., Ajani Y., Gohari A. R. (2015). Alpha-amylase inhibitory activity and phytochemical study of Zhumeria majdae Rech. F. and Wendelbo. *Pharmacognosy Research*.

[43] Khan U. A., Rahman H., Niaz Z. (2013). Antibacterial activity of some medicinal plants against selected human pathogenic bacteria. *European Journal of Microbiology and Immunology*.

[44] Bhatt B. D., Dahal P. (2019). Antioxidant and antimicrobial efficacy of various solvent extracts of seed of rudrakshya (Elaeocarpus ganitrus) from Ilam district of Nepal. *Journal of Nepal Chemical Society*.

